# Calcium, magnesium, and vitamin D supplementations as complementary therapy for hypertensive patients: a systematic review and meta-analysis

**DOI:** 10.1186/s12906-025-04809-x

**Published:** 2025-03-05

**Authors:** Samar A. Amer, Dina Essam Abo-elnour, Abdallah Abbas, Abdelrahman Salah Abdelrahman, Hossam-Eldin Mohamed Hamdy, Samar Kenawy, Menna M. Sarhan, Omar Hany Mohamed, Mohamed Yousif Elnaghy, Mohammed Baker, Rawan Medhat El-Gayar, Omnia Samy El-Sayed, Moamen Mostafa Asla

**Affiliations:** 1 Department of Public Health and Community Medicine, Faculty of Medicine, Zagazig University, Zagazig, Egypt; 2 Faculty of Medicine, Zagazig University, Zagazig, Egypt; 3 Faculty of Medicine, Al-Azhar University, Damietta, Egypt; 4 Faculty of Medicine, Alexandria University, Alexandria, Egypt; 5 Faculty of Medicine, Jordan University of Science and Technology, Irbid, Jordan

**Keywords:** Hypertension, Calcium, Magnesium, Vitamin D, Complementary therapies, Supplementation, Pulse rate

## Abstract

**Background:**

Hypertension, the first global modifiable risk factor for cardiovascular disease (CVD) morbidity and mortality, is a consequential and remediable threat to the health of individuals and society. Therefore, we conducted this study to explore the role of calcium (Ca^++^), magnesium (Mg^++^), and vitamin D (Vit-D) supplementation as complementary therapies for hypertension, focusing on their effects on systolic blood pressure (SBP), diastolic blood pressure (DBP), and pulse rate.

**Methods:**

This systematic review and meta-analysis examined relevant 6509 articles in PubMed, Scopus, Web of Science, and Cochrane CENTRAL up to October 2024. The primary outcome was the difference in blood pressure measurements (systolic and diastolic) and the pulse rate. The extracted data were analyzed using Open Meta Analyst software.

**Results:**

This systematic review and meta-analysis included 40 studies; of them, 24 studies were analyzed. Ca^++^ was associated with a significant drop in the DBP (MD: -2.04, 95% CI [-3.39, -0.69], *P* = 0.01), but not in the SBP (*P* = 0.34) or pulse rate (*P* = 0.84). Mg^++^ significantly reduced DBP (MD: -1.64, 95% CI [-3.19, -0.09], *P* = 0.04), but had no significant effect on the SBP (*P* = 0.16) or pulse rate (*P* = 0.81). The estimated effect of Vit-D showed a significant reduction in SBP (MD: -2.83, 95% CI [-5.47, -0.199], *P* = 0.04) and DBP (MD: -1.64, 95% CI [-2.97, -0.3], *P* = 0.01).

**Conclusion:**

Ca^++^ and Mg^++^ significantly reduced DBP but had no significant effect on SBP or the pulse rate. Whereas, vitamin D significantly reduced SBP and DBP.

**Supplementary Information:**

The online version contains supplementary material available at 10.1186/s12906-025-04809-x.

## Introduction

According to the 2023 World Health Organization (WHO) report, 1 in 3 persons had hypertension (silent disease) [1], defined as systolic blood pressure (SBP) ≥ 140 mmHg and/or diastolic blood pressure (DBP) ≥ 90 mmHg [2]. Globally, for example, the prevalence of hypertension among adults was higher in low- and middle-income countries (LMIC) (31.5%, 1.04 billion people) than in high-income countries (28.5%, 349 million people). However, from 1990 to 2015, the estimated number of BP-related all-cause and cardiovascular disease (CVD) deaths significantly increased; this is a leading preventable risk factor for all-cause CVD mortality [3, 4].

Sociodemographic, environmental, and behavioral factors are likely to explain racial and ethnic disparities in mean blood pressure and hypertension prevalence. Other modifiable risk factors for hypertension include alcohol, obesity, an unhealthy diet high in sodium and low in potassium, a lack of physical activity, air pollution, psychological stress, sleep disorders, and noise exposure [5–11].

The global mean BP has stayed steady or slightly dropped during the past four decades due to the widespread usage of antihypertensive drugs, which doubled to 1.3 billion from 650 million between 1990 and 2019 [1, 4]. Despite investing $216 million in primary health care to enhance hypertension care, 4 out of 5 hypertensive cases remain untreated. Treatment could save 76 million BP-related deaths between 2023 and 2050 [1, 12].

Primary care should offer improved hypertension treatment programs because the economic benefits outweigh the costs by an 18 to 1 ratio. Combining antihypertensive medication with additional nutrients and dietary supplements(DSs), such as the Dietary Approaches to Stop Hypertension (DASH) I and II diets, can have additive or synergistic benefits [2, 13, 14]. DSs, such as calcium (Ca^++^), magnesium (Mg^++^), and vitamin D, are a subgroup of complementary and alternative medicine (CAM), which the public commonly uses and touts as natural ways to affect blood pressure [14–16].

Ca^++^, a structural mineral required to contract muscles and signal cells, is abundant in salmon, soybeans, kale, cheese, and yogurt. BP regulation by Ca^++^ is unknown. Most studies found an inverse relationship between BP and Recommended Dietary Allowance (RDA) Ca^++^ intake (1000–1300 mg/day). Others found minimal or no hypotensive impact from Ca^++^ ingestion in normal and hypertensive subjects. Therefore, the recommendation was to combine Ca^++^ intake (1300 mg/day) with DASH to reduce the risk of hypertension [17, 18].

Oily fish, egg yolks, and red meat contain fat-soluble vitamin D (Vit-D). Dietary vitamin D becomes active vitamin D3 when exposed to sunlight. It maintains musculoskeletal, cardiovascular, and neurological health by controlling calcium homeostasis. Numerous studies have established an inverse link between Vit-D (or its metabolites, or its active form, Vit D3) and blood pressure and its subsequent consequences, such as CVD. Few trials showed no or slight hypotensive effects. Excess Vit-D can cause renal failure, stiffness (hypercalcemia), and vascular resistance. A few studies found that Vit-D and calcium had better hypotensive activity than vitamin D alone or calcium alone [19–22].

Magnesium (Mg^++^), “nature’s physiological calcium channel blocker,” is a cofactor for over 300 enzyme systems, including delta-6-desaturase. This enzyme converts linoleic acid to gamma linoleic acid. This is the longest stage in generating prostaglandin E (PGE1), which relaxes blood arteries and prevents platelet formation. Many foods contain it, especially nuts, unpolished grains, and leafy greens. Hypertension, cerebral and coronary vasospasms, and muscle cramps can result from magnesium shortage. For every 100 mg of dietary Mg^++^, ischemic stroke risk decreased by 8%. Hypertensive patients have altered Mg^++^ transporter TRPM7 channels [23–27].

The consumption of DSs as supplemental therapy differs by region, which may explain hypertension trends and incidence worldwide. Thus, lifestyle interventions targeting these risk factors may reduce worldwide inequities, hypertension prevalence, and BP [28]. Researchers have studied complementary therapies for blood pressure management, while pharmaceuticals remain beneficial. Numerous studies have demonstrated the lowering of blood pressure in hypertensives by Ca^++^, Mg^++^, and Vit-D supplements. Some investigations suggest the opposite hypothesis [29].

The aim of this study was to explore the role of Ca^++^, Mg^++^, and Vit-D supplementation as complementary therapies for hypertension, focusing on their effects on systolic blood pressure (SBP), diastolic blood pressure (DBP), and pulse rate.

## Methods

This systematic review and metanalysis followed the updated PRISMA 2020 guidelines and all steps reported in the Cochrane Handbook of Systematic Reviews [30, 31].

### Eligibility criteria

This study considered the retrieved articles eligible if they conformed to our PICOST (Population, Intervention, Control, Outcomes, Study Design, and Timeframe) framework. P: hypertensive patients; I: complementary therapy (Ca^++^, Mg^++^, and Vit-D); C: placebo; O: outcome, including SBP, DBP, and pulse rate; S: clinical trials in English, excluding review articles, unpublished manuscripts, conference abstracts, and book chapters; and T: from inception up to October 2024.

### Information sources and search strategy

A well-developed systematic search was performed through four online databases: PubMed, Scopus, Web of Science, and Cochrane CENTRAL. We looked for eligible articles up to October 2024 using the following search query: [(“complementary therap*” OR “alternative therap*” OR “complementary medicine” OR “alternative medicine” OR “supplementary therap*” OR “Vitamin D” OR “Calcitriol 24-Hydroxylase” OR “1 alpha,25-Dihydroxycholecalciferol-24-Hydroxylase” OR Cholecalciferol OR calcium OR magnesium) AND (hypertension OR hypertensive OR “high blood pressure” OR “elevated blood pressure”) AND (“clinical trial” OR “randomized controlled trial” OR RCT)]. A manual search was performed to examine the reference list of the included articles and related review articles.

### Selection process

We retrieved the search results from the databases using EndNote software. After duplicates removal, we extracted the articles into Rayyan software to start the screening process [32]. Four authors in two groups independently screened the title and abstracts of the resulting studies based on the eligibility criteria. The senior author, when necessary, resolved any disagreement through discussion. Then, the same authors independently reviewed the full texts of the included articles. Any disagreement was resolved by consulting the senior author.

### The data collection process

Four authors in two groups independently extracted the matched data using a well-developed Excel sheet. The senior author scrutinized the extracted data and resolved any disagreements through discussion. The extracted data were as follows: (1) Baseline characteristics, such as sample size, age, and sex; (2) A summary of the included article, detailing the design, sample sizes, health state, and dose and duration of treatment; and (3) Outcomes of interest, including blood pressure measurements and pulse rate.

### Quality assessment

Two authors independently assessed the quality of the included articles using the Cochrane risk of bias assessment tool for randomized controlled trials. Finally, they classified RCTs as having a high, unclear, or low risk of bias [33] according to the following domains: randomization process, allocation concealment, blinding of participants and personnel, blinding of outcome assessment, incomplete outcome data, selective reporting, and other bias. Any disagreement was resolved by discussion with the third senior author.

### Data synthesis

The extracted data were analyzed using Open Meta Analyst software [34]. We pooled continuous data using mean differences. We considered the data statistically significant if the *P*-value was < 0.05 and statistically heterogeneous if the *P*-value of the chi-square test was < 0.05 and the I-square test was > 60% [35]. A fixed-effect model was used except for the pooled heterogeneous studies, where a random-effect model was used. A sensitivity analysis was performed to identify the source of heterogeneity among the included studies. Because all the assessed outcomes included < 10 studies, we did not perform Egger’s test of publication bias [36].

## Results

### Study selection

**Fig. 1:**
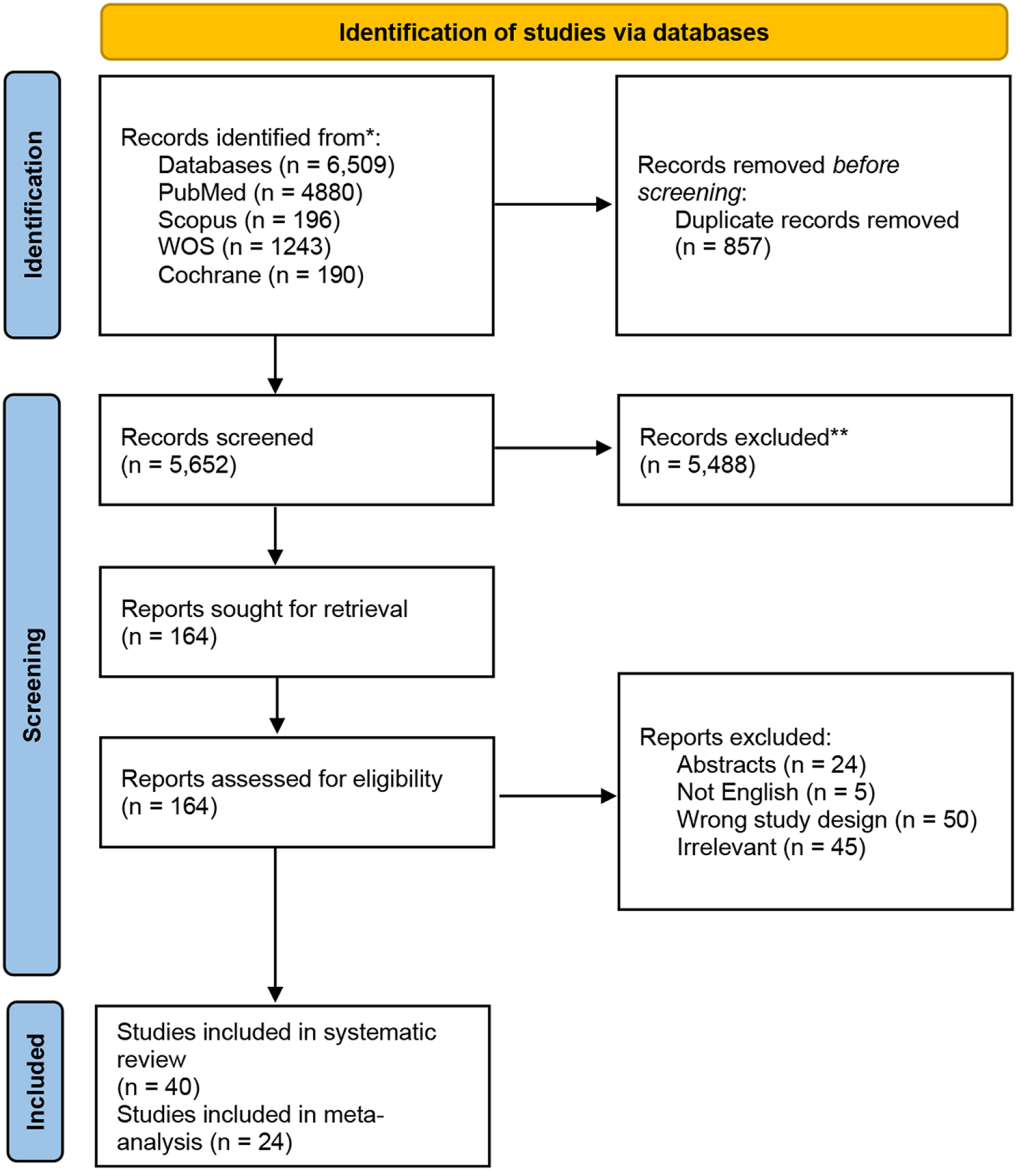
The PRISMA flow chart of selection process

Our database search resulted in 6509 records, which were reduced to 5652 after removing the duplicates. Following title and abstract screening, 164 studies were assessed for eligibility in full-text screening. Finally, the total number of included studies reached 40 studies, including 24 studies for meta-analysis. Figure 1 represents the PRISMA flow chart of the selection process.

### Study characteristics

The selected articles included a total of 2979 participants. Most of them had mild to moderate hypertension at the time of recruiting. Their ages ranged between 18 and 82 years old. We classified the included articles into three groups based on the intervention (Ca^++^, Mg^++^, and Vit-D). The included articles were designed as controlled clinical trials, either randomized controlled or crossover trials. The follow-up duration varied between only one week, as in Gonçalves 2020 [37], and one year and a half, as in Sluyter 2017 [38]. Table 1 represents a summary of the included articles.

**Table 1:** A summary of the included articles

Study ID	Design	Duration (wks)	Population	Sample size	Intervention group	Dose	*N*	Control group	*N*
Dazai 1994 [72]	Interventional	2	Mild to moderate HTN / stage I, II essential HTN	14	Ca^++^	7.7 g	14	placebo	5
de Paula 2020 [73]	RCT	8	HTN with diabetes	43	Vit-D	100,000 IU	22	pacebo	21
Ferrara 1992 [74]	Clinical trial	24	Mild to moderate primary HTN	14	Mg^++^	15mmol/day	7	placebo	7
Goncalves 2020 [37]	Pilot study	1	Hypertensive elderly women	11	Vit-D	Single dose 200.000 IU	11	placebo	5
Grobbee 1986 [75]	RCT	12	Mild HTN	90	Ca^++^	4-7gm	46	placebo	44
Guerrero-romero 2008 [52]	RCT	16	Diabetic, HTN on captopril or single drug	79	Mg^++^	2.5 g	40	placebo	39
Hatzistavri 2009 [51]	RCT	12	Uncomplicated HTN	48	Mg^++^	600 mg	24	placebo	24
Sanjuliani 1996 [39]	RCTCrossover	6	Mild to mederate HTN	15	Mg^++^	600 mg	15	placebo	15
Sheikh 2020 [76]	RCT		Essential HTN	208	Vit-D	1000U	104	placebo	104
						50,000U			
Sluyter 2017 [38]	RCT	1.5 year	Adult men and women aged 50 to 84 years and resident in Auckland, New Zealand.	517	Vit-D	200 000 IU (initial dose) followed 1 month later by monthly 100 000-IU doses	256	placebo	261
Cappuccio 1987 [77]	RCT Cross over	4	Mild to moderate essential HTN	18	Ca^++^	40 mmol/day or 1600 mg/day	18	placebo	18
Chen 2014 [78]	RCT	24	HTN Grade: 1&2	126	Vit-D	2000 IU/d	63	placebo	63
Cunhaa 2016 [79]	RCT	24	HTN women	35	Mg^++^	600 mg/day	17	placebo	18
Widman 1993 [80]	RCT Cross over	21	HTN patients	17	Mg hydroxide	15 mmol/day	17	placebo	17
Weinberger 1993 [81]	RCT Cross over	8	Normotensive and hypertensive	27	Ca^++^	1.5 g/day for 8 weeks	27	placebo	27
Witham 2014 [82]	RCT	24	HTN patients	68	Vit-D	100,000 U oral every 2 months	34	placebo	34
Witham 2013 [83]	RCT	48	Elderly with isolated systolic HTN and vit D levels < 30 ng/mL	159	Vit-D Group	of 100 000 U oral every 3 months for 1 year	80	placebo	79
Zemel 1990 [84]	RCT	12	Mild HTN	13	Mg^++^	40 mmol	7	placebo	6
Zhou 1994 [44]	RCT	14	HTN patients	57	Ca^++^	1000 mg/day	44	placebo	38
Zoccal 1988 [85]	RCT Cross over	8	Mild to moderate essential HTN	23	Ca^++^	1 g/d for 8 weeks orally	23	placebo	23
McCarron 1985 [86]	RCT Cross over	16 w and 4 w washout	48 hypertensive and 32 normotensive	80	Ca^++^	1 g	80	placebo	80
Meese 1978 [87]	RCT Cross over	8 w and 2 w washout	Uncomplicated 1ry HTN	41 began the study / 28 completed it	Ca^++^	800 mg/day	35	placebo	17
Kawano 1998 [88]	RCT Cross over	16	Untreated or treated hypertensive patients	60	Mg^++^	20 mmol/d	60	placebo	60
Mozaffari-Khosravi 2014 [40]	RCT	8	Patients with elevated BP and vit D deficiency	42	Vit-D	50 000 IU/week	19	oral liquid paraffin	20
Nowson 1989 [89]	RCT	8	Untreated, mild hypertensive subjects	25	Mg^++^	10 mmol/day	12	placebo	13
Nowson 1988 [90]	RCT Cross over			12	Ca^++^	20 mmol/day	12	placebo	12
Nowson 1989 [91]	RCT		Forty-seven patients with mildhypertension HTN) and 48 normotensive patients	95	Ca^++^	10 mmol/day	31	placebo	33
						20 mmol/day	31		
Larsen 2012 [92]	RCT	20	Hypertensive patients residing in Denmark	130	Vit-D	75 µg/day (3,000 IU)	55	placebo	57
Lasaridis 1989 [93]	RCT Cross over	10 days	Patients with uncomplicated essential HTN	18	Ca^++^	1 g	9	placebo	9
Pikilidou 2009 [42]	RCT	8	Patients with type 2 DM and HTN	31	Ca^++^	1,500 mg/d	15	placebo	16
Pilz 2015 [41]	RCT		Participants with arterial hypertension and 25-hydroxyvitamin D levels below 30 ng/mL.	200	Vit-D	2800 IU/d	100	placebo	100
Lind 1989 [94]	RCT		HTN	42	Alphacalcidol (Vit-D analogue)	1 µ%	39	placebo	
Barrios 2016 [95]	RCT	6	Essential HTN	45	Vit-D	1,000 IU daily	18	placebo	18
Cappuccio 1985 [96]	RCT	8	Mild to moderate essential HTN	17	Mg^++^	15 mmol Mg/day	17	placebo	17
Wimalawansa 1993 [97]	RCT	18	Mild to moderate essential HTN	8	Ca^++^	35 mmol/day	8	placebo	
Witteman 1994 [98]	RCT	24	Mild to moderate essential HTN	91	Mg^++^	20mmol/d	47	placebo	44
Theiler-Schwetz 2020 [99]	RCT	.	Adults with arterial HTN and a 25(OH)D serum concentration < 30 ng/Ml	200	Vit-D	2800 IU/day	100	placebo	100
Morris 1991 [100]	Clinical trial	48 w CaCO3, followed by 12 w placebo	Volunteers 50–80 years with SBP (when not taking antihypertensive medication) was consistently > 140 mmHg or if DBP was > 90 mmHg during a 4-week baseline period	128	Ca^++^	1 g/d	103	placebo	12
Lind 1991 [101]	RCT	24	Adults with DBP > 95 mmHg or DBP 85 to 94 mmHg together with SBP > 165 mmHg and without antihypertensive medication	71	Mg^++^	15 mmol	49	placebo	22
Santos 2024 [102]	RCT	24	Age- and gender-matched adults with obesity-related hypertension and vitamin D deficiency	36	Cholecalciferol		18	Placebo	18

### Quality assessment

According to the risk of bias assessment tool, we assessed the included studies as high risk, low risk, or some concerns. Most of the included studies fell into the category of low risk (*n* = 11) or raised some concerns. Four of the included studies were scored as high risk of bias. The supplementary table presents the quality assessment of the included articles.

### The effect of Ca^++^ on blood pressure and pulse

**Fig. 2:**
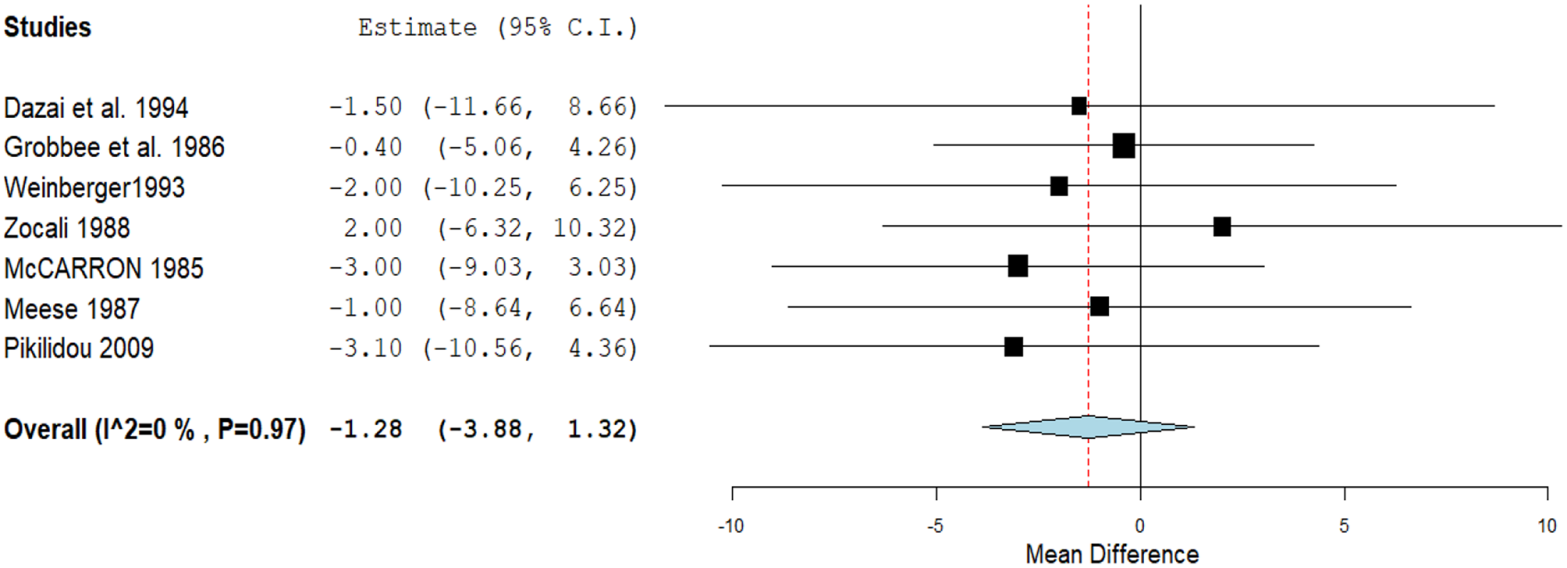
A forest plot of the effect of calcium on systolic blood pressure

In seven studies, Ca^++^ was found to have a non-significant effect on lowering SBP (MD: -1.28, 95% CI [-3.88, 1.32], *P* = 0.34). Pooled studies were homogenous (*P* = 0.97, I^2^ = 0%) (Fig. 2).

**Fig. 3:**
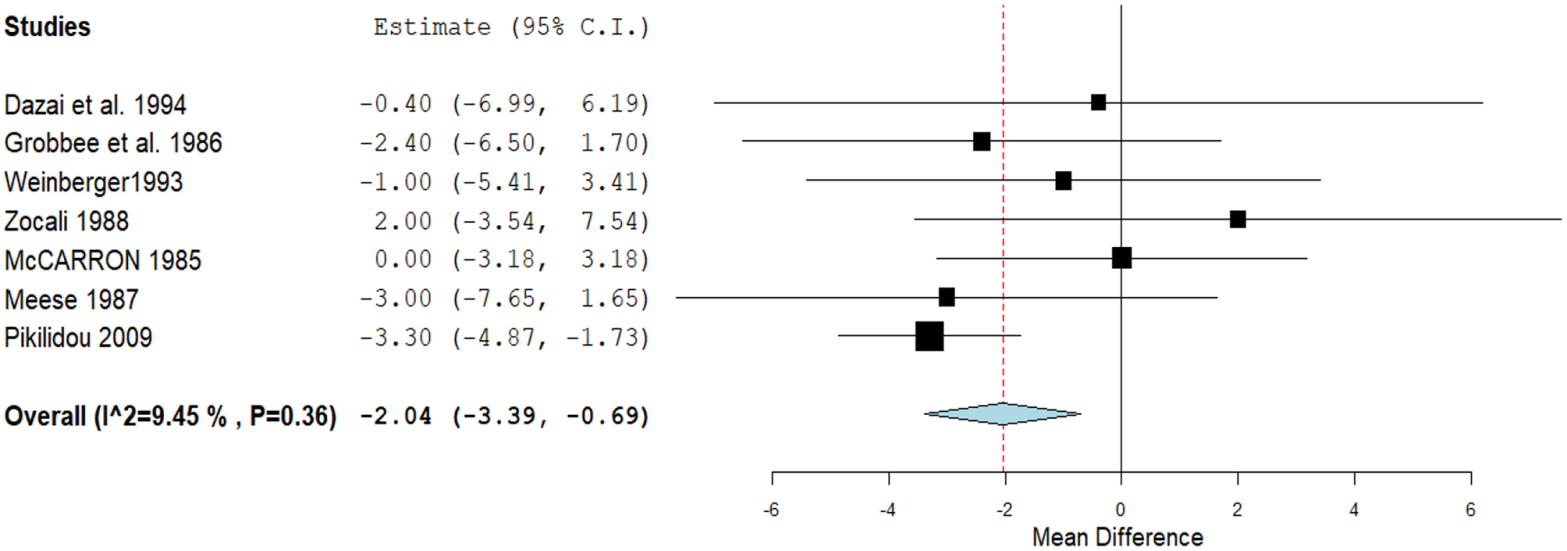
A forest plot of the effect of calcium on diastolic blood pressure

**Fig. 4:**
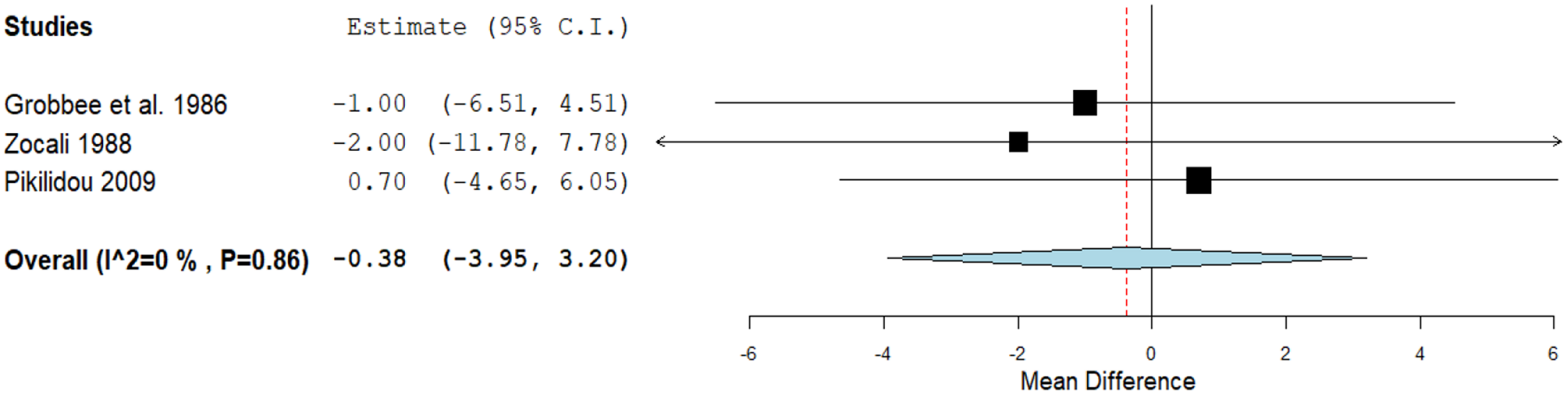
A forest plot of the effect of calcium on pulse rate

Seven studies achieved a statistically significant reduction in DBP. (MD: -2.04, 95% CI [-3.39, -0.69], *P* = 0.01). Pooled studies were homogenous (*P* = 0.36, I² = 9.4*%).* (Fig. 3). A non-significant decrease in the pulse rate was detected through three studies (MD: -0.38, 95% CI [-3.95, 3.2], *P* = 0.84). Pooled studies were homogenous (*P* = 0.86, I² = 0%) (Fig. 4).

### The effect of Mg^++^ on blood pressure and pulse rate

**Fig. 5:**
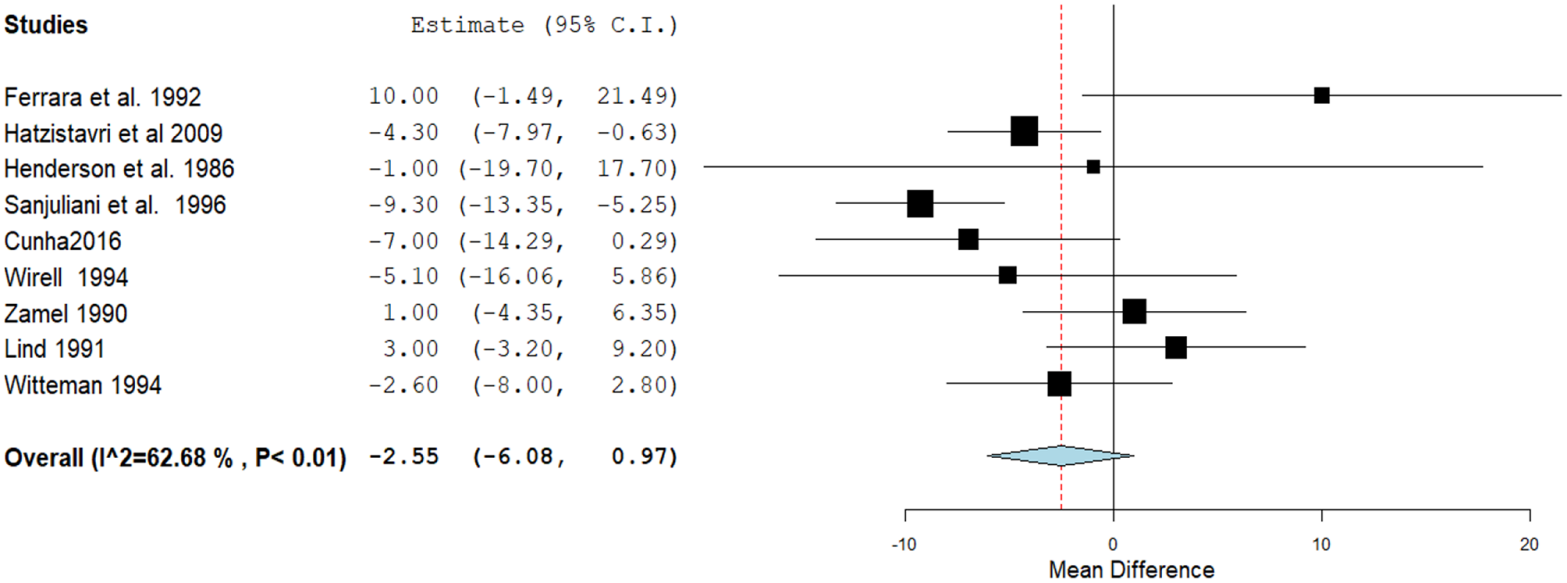
A forest plot of the effect of Magnesium on systolic blood pressure

Nine studies detected a non-statistically significant reduction in the SBP. The mean difference was − 2.55, with a 95% confidence interval of -6.08 to 0.97, and a *P* value of 0.16. Pooled studies were heterogeneous (*P* < 0.01, I² = 62.6%) (Fig. 5). Heterogeneity was best resolved by excluding Sanjuliani et al.‘s 1996 [39] without (*P* = 0.11, I^2^ = 39.4%) altering the results.

**Fig. 6:**
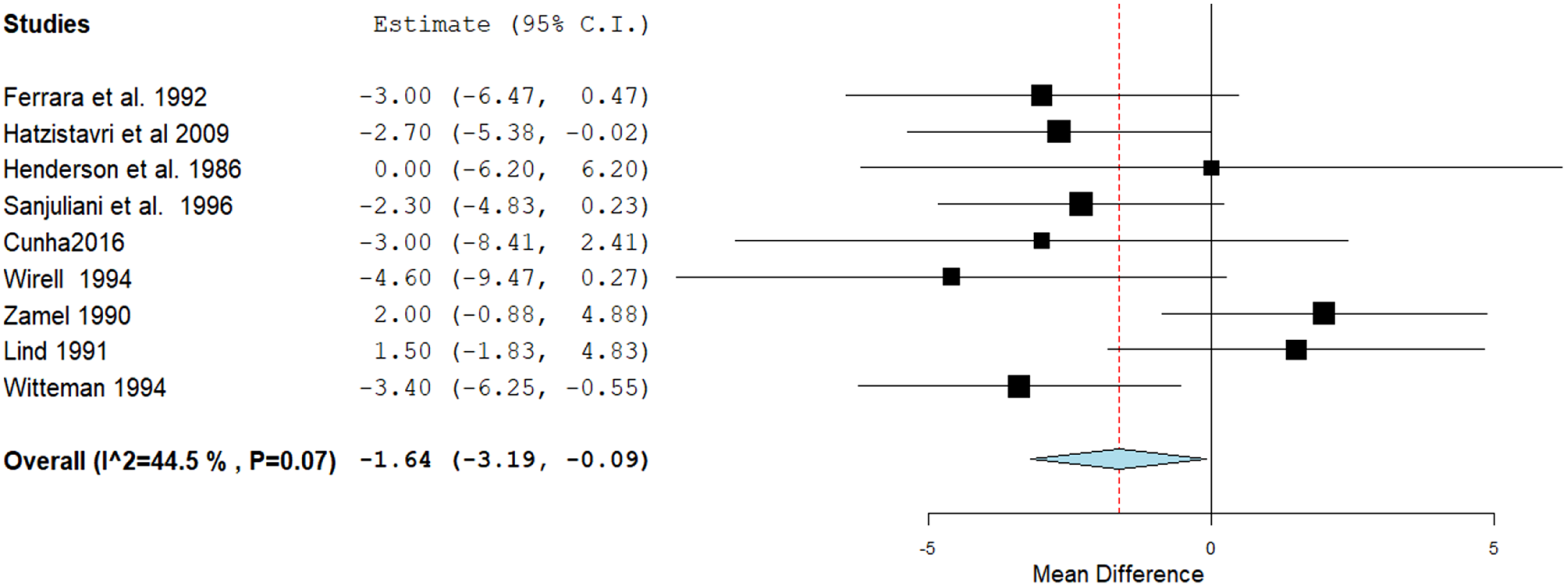
A forest plot of the effect of Magnesium on diastolic blood pressure

The estimated effect showed a statistically significant reduction in the DBP through nine studies (MD: -1.64, 95% CI [-3.19, -0.09], *P*= 0.04). Pooled studies were homogenous (*P* = 0.07, I² = 44.5%) (Fig. 6).

**Fig. 7:**
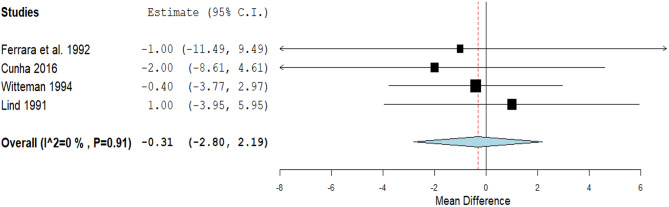
A forest plot of the effect of Magnesium on pulse rate

A non-significant decrease in the pulse rate was detected through four studies (MD: -0.31, 95% CI [-2.8, 2.19], *p* = 0.81). Pooled studies were homogenous (*P* = 0.91, I² = 0%) (Fig. 7).

### The effect of vitamin D on blood pressure

**Fig. 8:**
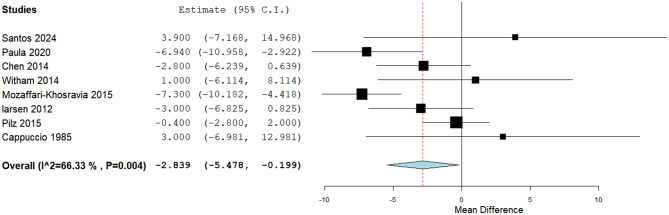
A forest plot of the effect of Vit-D on systolic blood pressure

The estimated effect showed a statistically significant reduction in the SBP through eight studies (MD: -2.84, 95% CI [-5.48, -0.199], *p* = 0.04). Pooled studies were heterogeneous (*P* = 0.004, I² = 66.3%) (Fig. 8). Heterogeneity was resolved by Mozaffari-Khosravia 2015 [40] exclusion (*P* = 0.08, I² = 47.9%); a significant difference was obtained (MD: -2.3, 95% CI [-4.7, -0.07], *p* = 0.057).

**Fig. 9:**
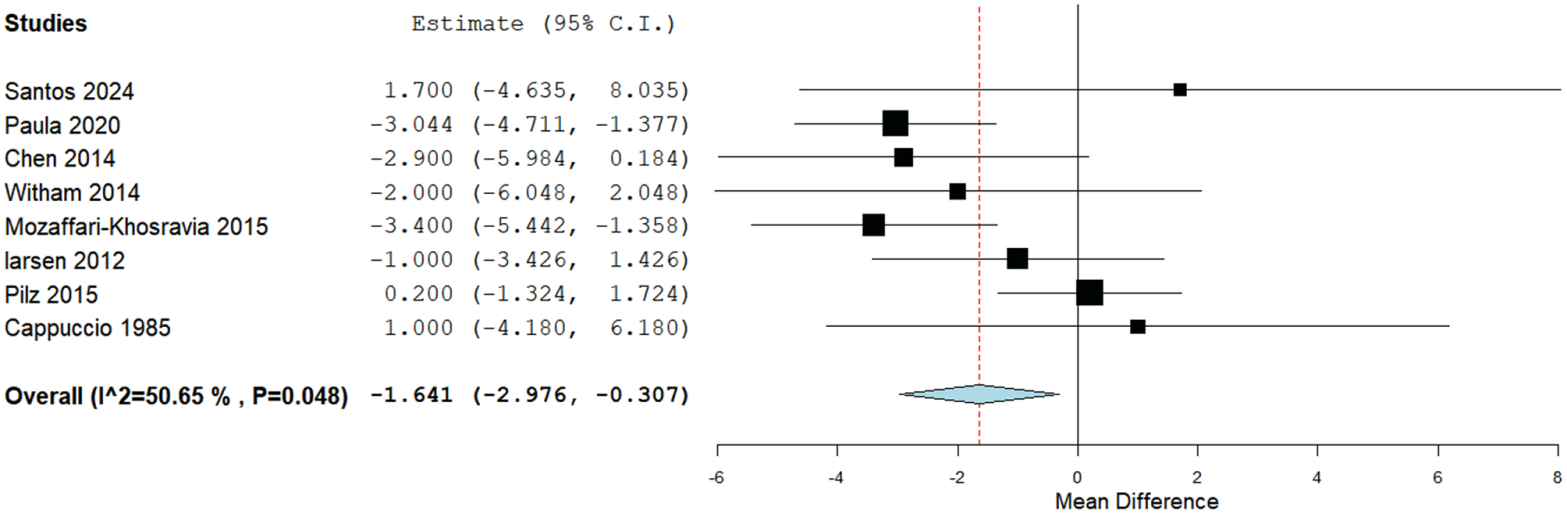
A forest plot of the effect of Vit-D on diastolic blood pressure

A statistically significant reduction in the DBP was detected through eight studies (MD: -1.64, 95% CI [-2.98, -0.3], *P*= 0.01). Pooled studies were heterogeneous (*P* = 0.04, I² = 50.6%) (Fig. 9). Heterogeneity was resolved by Pilz 2015 [41] exclusion (*P* = 0.48, I²=0%); no significant difference was obtained.

## Discussion

Hypertension is characterized by blood vessel damage due to vascular inflammation, structural remodeling, stiffer arteries, less flexibility, and loss of elasticity [2]. Therefore, etiologies exist for all non-mechanical causes, including metabolic, endocrine, nutritional, toxic, infectious, and others. Therefore, in this meta-analysis, we studied the effect of the following DS as complementary therapies in the management of hypertension and found that Ca^++^ significantly reduces DBP but has no significant effect on SBP or the pulse rate. Mg^++^ significantly reduces SBP and DBP but has no significant effect on the pulse rate. Vitamin D significantly reduces SBP and DBP.

### The effect of calcium supplementation on blood pressure and pulse

After comparing the SBP and DBP across 7 studies, this meta-analysis concluded that there was no statistically significant reduction in SBP (MD 1.28, 95% CI [3.88, 1.32], *p* = 0.34). After 8 weeks, Pikilidou et al. conducted a randomized control trial and found that the addition of Ca^++^ had no appreciable impact on the average ambulatory systolic and diastolic loads, pulse pressure, DBP, and SBP for the entire 24-hour day and night [42]. The belief that giving dietary Ca^++^ supplements to people with mild hypertension will lower blood pressure more effectively than a placebo is debunked [43]. However, Zhou et al.‘s RCT results demonstrate that oral Ca^++^ supplementation can significantly lower blood pressure in a significant number of essential hypertensive subjects [44]. Studies of the general population indicate a relationship between hypertension and calcium [45].

This is because Ca^++^ is a key part of smooth muscle contraction and cell signaling transduction. Also, the amount of Ca^++^ inside cells can control vascular tone, which makes vessels less constricted and more open. As a result, Ca^++^ directly affects blood pressure. Through controlling the SNS, Ca^++^ may improve the diuretic (Na^+^ excretion), control blood volume, and regulate cardiac output [45]. Moreover, reports suggest that Ca^++^ modifies the RAAS system, which in turn regulates the synthesis of AT-I and subsequently modifies blood pressure. Additionally, Ca^++^ indirectly controls BP, particularly by influencing parathyroid hormone secretion, which directly affects blood pressure [18, 46].

Compared to the DBP, we observed a significant decrease in it (MD 2.04, 95% CI [3.39, -0.69], *p* = 0.01).The study by Tanji et al. contradicts the finding by Zhou et al. that oral Ca^++^ supplementation significantly lowers BP [43]. Additionally, there was no statistically significant decrease in the pulse rate across the three studies (MD 0.38, 95% CI [3.95, 3.2], *P*= 0.84). while The study conducted by Pikilidou et al. indicated a lower pulse rate [42]. The different responses and inconsistent effects of calcium supplementation on blood pressure were because of the “ionic hypothesis” of high blood pressure, heart disease, and the metabolic, functional, and structural disorders that go along with them. Currently, we do not recommend calcium supplementation as an effective means to reduce BP [14, 45].

### The effect of magnesium supplementation on blood pressure and pulse

Regarding its effect on SBP, there is no significant effect. In line with a study by Lind et al., they did not find any evidence that Mg^++^ supplements worked for people with high-normal blood pressure or mild hypertension and do not generally recommend their use [14]. However, a 2009 study by Hatzistavri et al. indicates that taking oral Mg^++^ supplements may cause a slight but consistent drop in ambulatory BP in mild hypertension patients [47]. A meta-analysis of 22 trials involving 1173 patients revealed SBP reductions of 3–4 mmHg.

This can be attributed to many factors, including: (1) the effects are not as consistent as those observed with sodium (Na^+^) and potassium (K^+^); (2) the duration and the dose of Mg^++^ supplements. (3) In cases of renal insufficiency or co-medications that cause Mg^++^ retention, Mg^++^ supplements should be avoided or used cautiously. (4) The secondary causes of hypertension in a study by Banjanin that involved patients with essential hypertension, it was discovered that taking oral Mg^++^ supplements significantly reduced SBP and DBP [48–50].

***Regarding its effect on the DBP***, this meta-analysis from nine studies found that there was a statistically significant decrease in DBP (MD 1.64, 95% CI [3.19, -0.09], *p* = 0.04). This is in line with a 2009 study, which discovered that giving adults with diabetes and high blood pressure Mg^++^ supplements with MgCl₂ significantly reduced their SBP and DBP [51]. Furthermore, Yamamoto et al. found that supplements containing calcium and Mg^++^ are unlikely to lower BP in adults with high-normal DBP [52]. Numerous epidemiologic studies show a negative link between high blood pressure and eating a lot of Mg^++^ (at least 500–1000 mg/d), with a maximum drop of 5.6/2.8 mmHg. For example, BP dropped significantly after eight weeks of taking Mg^++^ supplements in 60 patients with essential hypertension. Increased Mg^++^ intake, along with high K^+^ and low Na^+^ intakes, or with the addition of taurine at a dose of 1000–2000 mg/d, enhances the Mg^++^ anti-hypertensive effects [48, 53].

Mg^++^ statistically significant decrease in DBP, this can be explained. Mg^++^ acts as a direct vasodilator, similar to a Calcium Channel Blockers (CCBs), as it competes with Na^+^ for binding sites and functions in vascular smooth muscle. Lowering oxLDL, HS-CRP, TBxA2, A-II, and norepinephrine and increasing PGE are some of the things that magnesium does. It also controls calcium, sodium, potassium, and pH levels inside cells and increases nitric oxide. It also enhances endothelial function. The systemic vascular resistance index and left cardiac work index both show improvements. Additionally, Mg^++^ improves glucose, insulin resistance, and MS. It also binds to potassium in a cooperative manner to reduce EDV and BP [48, 53–56].

Regarding its effect on the pulse rate, this meta-analysis of three studies found no statistically significant decrease. This is because Mg^++^ blocks NMDA receptors, regulates CCBs, maintains mitochondrial calcium levels, stops ischemia-induced glutamate release, and widens cerebral arteries. These actions lower cholesterol, stop the production of cytokines, stop nuclear factor Kb, lower oxidative stress, and stop platelets from sticking together to prevent thrombosis. Reductions in CVD and cardiac arrhythmias demonstrate these benefits; some suggested mechanisms include carotid IMT, cholesterol, and cytokine production [23–26, 57–61].

### The effect of vitamin D supplementation on blood pressure and pulse rate

This meta-analysis of seven studies that used vitamin D found that both SBP and DBP went down statistically significantly (MD 3.16, 95% CI [5.84, -0.48], *P*= 0.02) and (MD 1.77, 95% CI [3.14, -0.41], *P*= 0.01). Many meta-analyses (MAs) from different cross-sectional studies in 2011 agreed that there is an inverse relationship between the amount of vitamin D in the blood and HT. Another MA from eight prospective studies in 2015 also found an inverse relationship between the amount of vitamin D (25-hydroxyvitamin D) in the blood and the risk of HT [40]. What a 2015 study showed was that giving 50,000 IU of vitamin D by mouth once a week for 8 weeks to people who were vitamin D deficient could help prevent the deficiency, work with blood pressure medicines, and keep SBP, DBP, and MAP in check [62]. However, a MA found in 2016 that a daily Vit D3 intake (dose > 800 IU/day) for more than 6 months could significantly lower SBP and DBP in both hypertension and normotensive patients [63]. In 2019, MA concluded that oral vitamin D3 consumption significantly reduced both SBP and DBP in subjects with hypertension and vitamin D deficiency [64].

This is understandable given that vitamin D controls parathyroid hormone secretion as well as calcium homeostasis, specifically calcium absorption and metabolism, by acting on voltage-dependent calcium channels and RAAS directly through renin production. As a result, co-supplementing calcium and vitamin D demonstrated improved blood pressure regulation. Vitamin D also enhances endothelial function by reducing vascular resistance, calcification, and the inflammatory response. This, in turn, increases the production of Nitric oxide and maintains the tone of the vessels. Therefore, vitamin D helps suppress complications related to CVD [65–67].

In contrast, many MAs; in 2009, vitamin D was found to significantly lower DBP (− 3.1 mm Hg) in hypertensive subjects but not to significantly change mean BP or SBP. This showed modest hypotensive activity in hypertensive patients but no changes in normotensive subjects [66]. In 2010, researchers found that oral vitamin D supplementation lowered SBP but not DBP [67]. But in 2015, a meta-analysis from 46 RCTs [68] found that there is no evidence of a BP-lowering effect, while another meta-analysis in 2020 discovered that vitamin D supplementation did not reduce BP in the general population [69].

The analysis must consider certain limitations. The study employed a relatively small sample size, potentially diminishing the statistical power to identify a significant effect and encountered a high noncompliance rate; it excluded observational studies and uncontrolled trials. The included population was of variable ages. Some studies couldn’t find small differences between the intervention and placebo groups. The study also didn’t find a significant difference in blood pressure levels between subgroups with different follow-up times. However, whether vitamin D supplements can lower blood pressure for longer periods of time (> 2 years) remains unclear. Finally, the cohort studies conducted in Europe, America, and Asia may limit the generalizability of the results to other populations [66–69].

Therefore, to maintain normal blood levels of vitamin D, the Food and Drug Administration (FDA) and WHO recommended consuming 10–20 µg/day of the vitamin in oral supplementation, which would raise vitamin D3 levels, which have an inverse relationship with blood pressure. Adjuvant therapy, which combines vitamin D3 with other minerals or macronutrients, can further reduce blood pressure in hypertensive patients who are vitamin D deficient. You can take supplements containing 500–1000 mg of magnesium daily. Amino acid-chelated magnesium formulations have the potential to enhance absorption and reduce diarrheal episodes [70, 71].

However, this meta-analysis has many strengths, as it included a total of 2979 hypertensive patients from 40 studies after the two screening steps of 6509 studies from four databases, up to October 2024, to explore the effect of three essential supplements on three essential parameters: SBP, DBP, and pulse rate.

## Conclusion

This meta-analysis concluded that Ca^++^ significantly reduces DBP but has no significant effect on SBP or the pulse rate. However, Mg^++^ significantly reduces SBP and DBP but has no significant effect on the pulse rate. On the other hand, the meta-analysis for vitamin D supplementation revealed a statistically significant reduction in both SBP and DBP.

### Recommendations

- This meta-analysis suggests adding Mg^++^ and vitamin D supplements to hypertension therapy to lower SBP and DBP.
- Future studies should use larger, better-controlled RCTs with longer follow-ups and standardize the dose of these supplements to find out how they should be taken to get the best blood pressure effects. These studies should also look at the long-term effects of these supplements on blood pressure, CVD, and other comorbidities, as well as how they interact with other hypertension treatments.

By following these guidelines, future studies can confirm the benefits of calcium, magnesium, and vitamin D supplements for hypertension. This will improve patient outcomes and guide therapeutic practice.

## Electronic supplementary material

Below is the link to the electronic supplementary material.

Supplementary Material 1

## Data Availability

The datasets used and/or analyzed during the current study are available from the corresponding author (Dr_samar11@yahoo.com) on reasonable request.

## Abbreviations

BP: Blood pressure
Ca^++^: Calcium
CCBs: Calcium Channel Blockers
CVD: Cardiovascular disease
DBP: Diastolic blood pressure
DSs: Dietary supplements
LMIC: Low- and middle-income countries
Mg^++^: Magnesium
MA: Meta-analyses
K^+^: Potassium
RCTs: Randomized clinical trials
RDA: Recommended Dietary Allowance
Na^+^: Sodium
SBP: Systolic blood pressure
DASH: The Dietary Approaches to Stop Hypertension
WHO: World Health Organization
PGE: Prostaglandin E
Vit-D: Vitamin D
HTP: Hypertension
IU: International Unit
