# Supplementary material for: Calcium, magnesium, and vitamin D supplementations as complementary therapy for hypertensive patients: a systematic review and meta-analysis
Source: BMC Complement Med Ther. 2025 Mar 5;25:89. doi: 10.1186/s12906-025-04809-x (PMC11884002; doi:10.1186/s12906-025-04809-x)
Supplement: Supplementary file 1 — Supplementary Material 1 [file 12906_2025_4809_MOESM1_ESM.docx]

**Supplementary Table: Risk of bias assessment**

| Study ID | D1 | D2 | D3 | D4 | D5 | D6 | D7 | Overall |
| --- | --- | --- | --- | --- | --- | --- | --- | --- |
| Barrios 2016 | Low | Low | Low | Unclear | Low |  | Unclear | Some concern |
| Cappuccio 1985 | Unclear | Low | Low | Low | Low | Unclear | Unclear | Some concern |
| Cappuccio 1987 | Unclear | Unclear | Low | Low | Low | Unclear | Low | Some concern |
| Chen 2014 | Low | Low | Low | Low | Low | Low | Low | Low |
| Cunha 2016 | Unclear | Low | Unlcear | Unclear | Unclear | Low | Low | Some concern |
| Morris 1991 | Unclear | Unclear | Low | High | High | Low | Low | High |
| Dazai 1994 | High | High | High | Low | Low | Low | Unclear | High |
| Ferrara 1992 | Unclear | Unclear | Low | Low | Low | Low | Unclear | Some concern |
| GONÇALVES 2020 | Unclear | High | Low | Low | Low | Low | Low | High |
| Grobbee 1986 | Unclear | Unclear | Low | Low | Low | Low | Low | Some concern |
| Guerrero-romero 2008 | Low | Low | Low | Low | Low | Low | Low | Low |
| Hatzistavri 2009 | Unclear | Unclear | High | Low | Low | Low | Low | Some concern |
| Kawano 1998 | Low | Low | Unclear | Unclear | Low |  | Low | Some concern |
| Lasaridis 1989 | Unclear | Unclear | High | High | Unclear | Low | Low | High |
| Lind 1989 |  |  |  | Unclear | Low | Unclear | Unclear | Some concern |
| Lind 1991 | Unclear | Unclear | Low | Low | Low | Unclear | Low | Some concern |
| Larsen 2012 | Low | Low | Low | Low | Low | Low | Low | Low |
| McCarron1985 | Low | Low | Low | Low | Low | Unclear | Low | Low |
| Meese 1978 | Unclear | Low | Low | Low | Unclear |  | Low | Some concern |
| Mozaffari-Khosravi 2014 | Low | Low | Low | Low | Low | Low | Low | Low |
| Nowson 1988 | Unclear | Unclear | Unclear | Unclear | Unclear |  | Unclear | Some concern |
| Nowson 1989 | Unclear | Unclear | Low | Low | Low |  | Low | Some concern |
| Nowson 1989 | Unclear | Low | Low | Low | Unclear | Unclear | Unclear | Some concern |
| Pikilidou 2009 | Low | Unclear | Unclear | Low | Unclear | Unclear | Unclear | Some concern |
| Pilz 2015 | Low | Low | Low | Unclear | Low | Unclear | Unclear | Some concern |
| Sanjuliani 1996 | Unclear | Unclear | Low | Low | Low | Low | Low | Some concern |
| Santos 2024 | Low | Low | Low | Low | Low | Unclear | Low | Low |
| Theiler-Schwetz 2020 | Low | Low | Low | Low | Low | Low | Low | Low |
| Sheikh 2020 | Low | Low | Low | Low | Low | Low | Low | Low |
| Sluyter 2017 | Low | Low | Low | Low | Low | Low | Low | Low |
| de Paula 2020 | Low | Low | Low | Low | Low | Low | Low | Low |
| Weinberger 1993 | Unclear | Unclear | Unlcear | Unclear | Low | Unclear | Low | Some concern |
| Widman 1993 | Unclear | Low | Low | Unclear | Low | Unclear | Low | Some concern |
| Witham 2014 | Unclear | Low | Low | Low | Low | Low | Low | Some concern |
| Witteman 1994 | Low | Unclear | Unlcear | Unclear | Low | Low | Low | Some concern |
| Witham 2013 | Low | Low | Low | Low | Low | Low | Low | Low |
| Wimalawansa 1993 | Unclear | Unclear | Unclear | Unclear | Unclear | Unclear | Unclear | Some concern |
| Zemel 1989 | Unclear | High | Unlcear | Unclear | Low | Unclear | Low | Some concern |
| Zhou 1994 | Unclear | Unclear | Unlcear | Unclear | Low | Low | Low | Some concern |
| Zoccal 1988 | Unclear | Unclear | Low | Low | Low | Low | Low | Some concern |

D1: randomization process, D2: allocation concealment, D3: blinding of participants and personnel, D4: blinding of outcome assessment, D5: incomplete outcome data, D6: selective reporting, D7: other bias
